# As I lie sleepless this night……

**DOI:** 10.4103/0019-5545.39766

**Published:** 2008

**Authors:** T. M. Raghuram

**Affiliations:** *Asst. Prof. of Psychiatry at M.E.S. Medical College, Perintalmanna, Kerala. E-mail:*tmraghuram@gmail.com

……….I remember and envy the hapless ones

who curl on pavements

sans blanket sans quilt

in the cold night and still

manage to snore away without a care;

……….I listen to the whispers

of the December wind

to the trembling leaves

and the cricket's musical messages,

to rue over my apologies

that fall in a heap

at my beloved's slumbering ear;

………..I listen to my own words boomerang,

hover persistent around the bedstead,

words that escaped my grasp

like mischievous moths

to taunt me, haunt me for life;

………..I revisit battle scenes

where I was the vanquished,

yet cringe in shame

at crimes uncommitted,

my sword of retribution

still in its scabbard rusting;

…………I savour those ephemeral ecstasies,

wishing it were endless

and begin planning, preparing

for greater ecstasies tomorrow;

…………I recall long lost souls,

their good deeds, loving smiles,

pledging to myself

atonement for forgetting them

just at the right moment;

As I lie sleepless for hours on end

counting the strokes of heartless time,

Slumber slowly envelopes my tired self

like a tearful mother pulling the sheets

over a punished child

sobbing in his sleep,

she, determined never to be punitive

and he, perhaps dreaming

never to hurt her again…..

